# Establishment of a Rabbit Oct4 Promoter-Based EGFP Reporter System

**DOI:** 10.1371/journal.pone.0109728

**Published:** 2014-10-31

**Authors:** Longquan Quan, Yongqiang Chen, Jun Song, Quanmei Yan, Quanjun Zhang, Sisi Lai, Nana Fan, Jige Xin, Qingjian Zou, Liangxue Lai

**Affiliations:** 1 Jilin Provincial Key Laboratory of Animal Embryo Engineering, College of Animal Science, Jilin University, Changchun, China; 2 Key Laboratory of Regenerative Biology, South China Institute for Stem Cell Biology and Regenerative Medicine, Guangzhou Institutes of Biomedicine and Health, Chinese Academy of Sciences, Guangzhou, China; Institute of Zoology, Chinese Academy of Sciences, China

## Abstract

Rabbits are commonly used as laboratory animal models to investigate human diseases and phylogenetic development. However, pluripotent stem cells that contribute to germline transmission have yet to be established in rabbits. The transcription factor Oct4, also known as Pou5f1, is considered essential for the maintenance of the pluripotency of stem cells. Hence, pluripotent cells can be identified by monitoring Oct4 expression using a well-established Oct4 promoter-based reporter system. This study developed a rabbit Oct4 promoter-based enhanced green fluorescent protein (EGFP) reporter system by transfecting pROP2-EGFP into rabbit fetal fibroblasts (RFFs). The transgenic RFFs were used as donor cells for somatic cell nuclear transfer (SCNT). The EGFP expression was detected in the blastocysts and genital ridges of SCNT fetuses. Fibroblasts and neural stem cells (NSCs) were derived from the SCNT fetuses. EGFP was also reactivated in blastocysts after the second SCNT, and induced pluripotent stem cells (iPSCs) were obtained after reprogramming using Yamanaka's factors. The results above indicated that a rabbit reporter system used to monitor the differentiating status of cells was successfully developed.

## Introduction

Embryon ic stem cells (ESCs) are unique cell populations with self-renewal, proliferating and pluripotent properties. ESCs have also been used to study developmental biology, genetic modification and regenerative medicine. Induced pluripotent stem cells (iPSCs) have been generated from several mammals and opened a new frontier in regenerative medicine [1]–[5]. Before ESCs or iPSCs are clinically applied to humans, extensive pre-clinical studies should be performed using suitable animal models to assess the safety, efficacy and long-term survival of these cells. Surgical operations can be performed more easily with rabbits (*Oryctolagus cuniculus*) as laboratory animal models than mice or rats because of rabbits' large size. Moreover, the physiology of rabbits is close to that of primates.

Previous studies found that rabbit ESCs (Rb-ESCs) and iPSCs (Rb-iPSCs) share important characteristics with human ESCs [2], [6]. However, germline-competent Rb-ESC lines have yet to be established. The lack of authentic ESCs restricts the availability of efficient transgenic technologies to develop genetically modified rabbits.

The transcription factor Oct4 is specifically expressed in pluripotent cells, such as inner cell mass (ICM), ESCs, primordial germ cells (PGCs) and embryonic carcinoma cells (ECs). Oct4 maintains the pluripotency of stem cells [7] and is used as one of the key transcript factors involved in the reprogramming of somatic cells into pluripotent cells [8]. Oct4 is also used as a classic pluripotent stem cell marker because the promoter of this transcription factor is highly active when cells are in a pluripotent state. Hence, an Oct4 promoter-based reporter system is a useful tool to monitor the differentiating status of cells in vivo and in vitro. Oct4-LacZ transgenic mice [9], Oct4-EGFP transgenic mice (OG2 mice) [10], murine Oct4-EGFP transgenic pigs [11], porcine Oct4-EGFP transgenic pigs [12] and OG2 human ESC lines [13] have been used to elucidate the early development of embryos, the mechanism of cell self-renewal and the differentiation and induction of pluripotent stem cells from somatic cells [14].

In this study, we established an Oct4 promoter-based EGFP reporter system to monitor pluripotent status of rabbit cells during nuclear transfer and induced reprogramming. This system can be used to optimise the nuclear transfer and the culture conditions for developing rabbit pluripotent stem cell lines.

## Materials and Methods

### Animals and Ethics statement

Rabbits used in the experiments were obtained from Center for Laboratory Animal Sciences of Southern medical University with an approval number SCXK (Guangdong) 2011–0015. The protocol was approved by the Institutional Animal Care and Use Committee of Guangzhou Institute of Biomedicine and Health, Chinese Academy of Sciences (ID 2010032) and the Department of Science and Technology of Guangdong Province (ID SYXK 2005–0063). Surgical procedures were performed under anesthesia according to guidelines of Institutional Animal Care and Use Committee of Guangzhou Institute of Biomedicine and Health, Chinese Academy of Sciences (Animal Welfare Assurance #A5748-01), and all efforts were made to minimize suffering.

### Construction of a Rabbit Oct4 Promoter-based EGFP Reporter vector

Similar to that of mice, humans and pigs, the Oct4 promoter of rabbits contains four conserved regions, including CR1, CR2, CR3 and CR4, and two enhancers, namely, DE and PE [15]. Rabbit Oct4 promoter region was amplified by polymerase chain reaction (PCR) with the following primers: RbOct4p-F: 5′-TTGAAATGGTTGCAATTGGAGGATATCC-3′ and RbOct4p-R: 5′-TCGGGGGTTTGCTCCAGCTTCTCCT-3′. The genomic DNA of New Zealand white rabbit was used as a template. The 3.0 kb DNA fragment was inserted into a pEGFP-N2 vector (#6081-1, Clontech) to produce pROP2-EGFP. The CMV promoter and the EGFP start codon ATG were replaced with the rabbit Oct4 promoter and the first exon, respectively. So, the protein products of pROP2-EGFP are fusion proteins, which contain the first exon of Oct4 gene in N-terminus and whole EGFP coding sites in C-terminus.

### Validity of pROP2-EGFP vector in rabbit embryonic stem cells (Rb-ESCs)

To generate Rb-ESCs, the day-4.5 blastocysts were flushed from rabbit uterus. After zona pellucida removed by mechanical separation, blastocysts were seeded on mitomycin C-treated MEF feeder layers in BL medium, including DMEM supplemented with 20% KnockOut Serum Replacement (KSR), 2 mM GlutaMax, 1% non-essential amino acids, 0.1 mmol/l β-mercaptoethanol, recombinant human bFGF (Sino Biological) and 10 ng/ml recombinant human leukemia inhibitory factor (LIF, Millipore). Approximately one week after seeding, ES colonies derived from ICM were mechanically separated into small clumps and reseeded on fresh feeder layers. After cultured for 2 passages, Rb-ESCs were used to test the validity of pROP2-EGFP vectors. Rb-ESCs were transfected with pROP2-EGFP vectors using Lipofectamine LTX reagent according to the manufacturer's protocol.

### Isolation and culture of rabbit fetal fibroblasts, neural stem cells

Cell culture reagents were purchased from Invitrogen except those that were specifically mentioned. RFFs were isolated from the fetuses of an 18-day-pregnant New Zealand white rabbit. After the head, viscera and extremities were removed, the tissues were minced into small pieces by using a scalpel blade and digested for 4 hours with collagenase (300 µg/ml, Sigma) and DNase (1.4 mg/ml, Sigma) at 37°C. An equal amount of Dulbecco's modified Eagle's medium (DMEM) containing 15% fetal bovine serum (FBS, Hyclone) was added and pipetted vertically to induce tissue dissociation. The dissociated cells were centrifuged at 300×*g* for 10 min and resuspended in RFF culture medium containing DMEM supplemented with 15% FBS, 2 mM L-glutamine, 0.1 mM sodium pyruvate and penicillin/streptomycin. The cells were cultured in 100 mm dishes. Place the dish in an incubator (37°C, 5% CO2 in air).

Rb-NSCs were isolated from the brains (meninges removed) of the fetuses obtained from an 18-day-pregnant rabbit. Rabbit brains were cut into small pieces and incubated in 0.25% Trypsin-EDTA for 15 min. Cell pellets were collected and rinsed twice with phosphate buffered saline (PBS). The cells were then cultured in a StemPro NSC SFM kit containing 20 ng/ml human bFGF and 20 ng/ml human EGF.

### Expression assay of reporter in mouse ESCs and RFFs

Mouse ES cell line R1 was cultured in mitomycin C-treated mouse embryonic fibroblast (MEF) feeder layers with an ES medium containing DMEM, 15% FBS, 2 mM GlutaMax, 1% non-essential amino acids, 0.1 mmol/L β-mercaptoethanol and 1000 units/ml ESGRO (Millipore). CMV-EGFP (pEGFP-N2), PGK-EGFP (PGK promoter replacing CMV promoter of pEGFP-N2), pROP2-EGFP and empty vectors were transfected into R1 cell lines with Xfect mESC Transfection Reagent (Clontech) and RFFs with Lipofectamine LTX reagent, respectively, according to the manufacturer's protocol.

### Stable transfection and genomic PCR

pROP2-EGFP vectors were linearized with the restriction enzyme Ase I and then transfected to RFFs by electroporation at 1350 V, 30 ms and 1 pulse number by using a Neon transfection system (Invitrogen). After 24 hours of culture, selective media containing 800 µg/mL G418 (Sigma) were added, and the cells were cultured for another 10 days. G418-resistant cell colonies were collected and then cultured for two to three passages in the succeeding 10 to 15 days. When the colonies multiplied to approximately 1×10^5^ cells, a part of each colony was collected and lysed in lysis buffer containing NP-40 (0.45%, Sigma) and proteinase K (1 mg/ml, TAKARA) at 56°C for 20 min and then at 90°C for 5 min. Cell lysates were used as a genomic template to detect the integration of exogenous genes by PCR. The used primers were ROPG-F: 5′-CGTATGACTTCTGCGGAGCG-3′ and ROPG-R: 5′-TGTAGTTGCCGTCGTCCTTGA-3′. Transgenic positive colonies were arrested at G0/G1 phase by inducing serum starvation for 4 to 6 days and used as donor cells for SCNT.

### SCNT

MII oocytes were collected from super-ovulated New Zealand rabbits as previously described [16]. Cumulus cells were removed from cumulus-oocyte complexes (COCs). Denuded oocytes were incubated in EBSS-complete medium. The metaphase plate and the polar body were visualised under an inverted microscope equipped with a Spindleview system (Cambridge Research & Instrumentation Inc.) and then removed using an enucleation pipette. Afterwards, donor cells at the G0/G1 phase were inserted into the perivitelline space of the enucleated oocytes. Oocytes were induced to undergo cell membrane fusion by applying three DC electrical pulses at 3.0 kV/cm for 20 µs in an activation medium [0.25 M sorbitol in water supplemented with 0.5 mM HEPES, 0.1 mM Ca(CH_3_COO)_2_, 0.5 mM Mg(CH_3_COO)_2_ and 1 mg/ml bovine serum albumin]. Reconstructed embryos were incubated for 1 hour in EBSS-complete medium at 38°C and then activated by applying the same electric pulses as used for fusion. The activated NT embryos were incubated for another 1 hour in an EBSS-complete medium containing 2 mM 6-dimethylaminopuriene and 5 µg/ml cycloheximide. After chemical activation, NT-embryos were cultured in the EBSS-complete medium at 38°C in a humidified atmosphere with 5% CO_2_. Several NT-embryos were cultured and developed in vitro until the blastocyst stage was reached. Other NT-embryos were transferred to recipients for further development after these embryos were cultured for 16 to 18 hours.

### Induction and maintenance of Rb-iPSCs

Rb-iPSCs were induced according to a previously described protocol [2]. In brief, human Oct4, Sox2, Klf4 and c-Myc were derived from human H9 ESCs by reverse transcription polymerase chain reaction (RT-PCR) and then cloned into different lentiviral vector FUGW (Addgene, 14883), namely, FUW-hOct4, FUW-hSox2, FUW-hKlf4 and FUW-hcMyc, respectively. These plasmids were packaged in a virus in 293T cell lines by co-transfecting with auxiliary packaging vectors (psPAX2 and pMD 2.G). Lentivirus were harvested and centrifuged at 80,000×*g* for 2 hours at 4°C in a SW28 swinging bucket rotor (Beckmann, USA). After centrifugation, the supernatant was carefully discarded and the pellets were suspended in Opti-MEM reduced serum medium at 4°C overnight.

Rb-NSCs were seeded at 1×10^4^ cells per well in a 12-well plate. On the next day, each concentrated virus was added to the medium with the MOI (MOI = viral titer/cell number) from 40 to 80 for each lentivirus and incubated for 24 h. Three days after the initial viral transduction, the cells were digested with Accutase (Sigma) and seeded onto MEF feeder layers. On day 4, two different ES media were used to replace RFF medium for further culture, respectively. The first one is called BL medium as previous described, which had been used to establish Rb-iPS previously [2]. The second one is called 3i medium, which had been used for establishment of both rat and mouse pluripotent cells [17], [18], and contained knockout DMEM supplemented with N2 and B27 supplements, 2 mM GlutaMax, 1% non-essential amino acids, 0.1 mmol/l β-mercaptoethanol, 10 ng/ml recombinant human LIF and three inhibitor molecules (1 mM FGF receptor inhibitor SU5402, 1 mM inhibitor of the MEK-activated PD0325901 and 3 mM GSK3 inhibitor CHIR99021). At 9 to 12 days, the Rb-iPS colonies were harvested and re-plated onto new plates for further culture.

### Karyotype analysis

Rb-iPSCs were cultured in 100 mm dishes for 2 days and incubated with 200 ng/ml colcemid for 2.5 hours. The cells were blown carefully, lysed with hypotonic buffer and fixed in acetic acid/methanol (vol/vol = 1∶3). Chromosomes at metaphase were stained with 5% Giemsa (Invitrogen) for 15 min. These chromosomes were photographed and analysed using Karyo 3.0 software.

### Tri-lineage Differentiation in vitro

The differentiation protocol used in this study was based on previously described methods [19]. In brief, Rb-iPSCs were grown as embryoid bodies (EBs) in DMEM/F12 media supplemented with 10% FBS for one week. To form mesoderm and endoderm lineages, EBs were then plated to 24-well culture dishes pre-treated with Matrigel (BD) to form a monolayer. To induce ectoderm cells, EBs were cultured in DMEM/F12 with 1% N2-supplemented retinoic acid (2 µM, Sigma) and forskolin (5 µM, Sigma).

### Gene expression analysis

Total RNA was extracted using a total RNA kit II (Omega) from different cells and tissues. First-strand cDNAs were then synthesised using a PrimeScript RT reagent kit (TAKARA) according to the manufacturer's instructions. PCR was performed using Premix Ex Taq Version 2.0 (TAKARA). The primer sequences used were as follows: GAPDH (sense: 5′-TGGCTACAGCAACAGGGTGGTG-3′, antisense: 5′-GGGGTCTGGGATGGAAACTGTG-3′); endogenous Oct4 (sense: 5′-CTTCCCCTCTGTGCCTGTCC-3′, antisense: 5′-AACTCCTTGCCCCACCCTTT-3′); and exogenous Oct4 (sense: 5′-CACTGTACTCCTCGGTCCCTTTC-3′, antisense: 5′-GCGTATCCACATAGCGTAAAAGG-3′).

### Immunofluorescent analysis

Cells or embryos were fixed with 4% paraformaldehyde fixing solution (PFA) for 15 min, washed with PBS and incubated in blocking buffer (5% goat serum, 1% BSA, 0.2% Triton-X 100 in PBS) for 45 min at room temperature. After the blocking buffer was removed, the cells were incubated overnight at 4°C with primary antibodies (5% goat serum and 1% BSA in PBS). The following antibodies were used: anti-Oct4 (1∶200, sc-5279, Santa Cruz); anti-β3 tubulin (1∶100, AT809, Beyotime); anti-α-Actin (1∶300, AA132, Beyotime); and anti-AFP (C19; 1∶100, sc-8108, Santa Cruz). On the following day, cells or embryos were washed with PBS. Afterwards, fluorescence-labelled secondary antibody (10% goat serum in PBS) 1∶300 Cy3 (A0521, Beyotime) or Alexa-594 (A21468, Invitrogen) was added and incubated in the dark at room temperature for 1 hours. The cells were washed and stained with DAPI solution (1 µg/ml).

## Results

### Construction and function validation of rabbit Oct4 reporter vector

**Figure 1:**
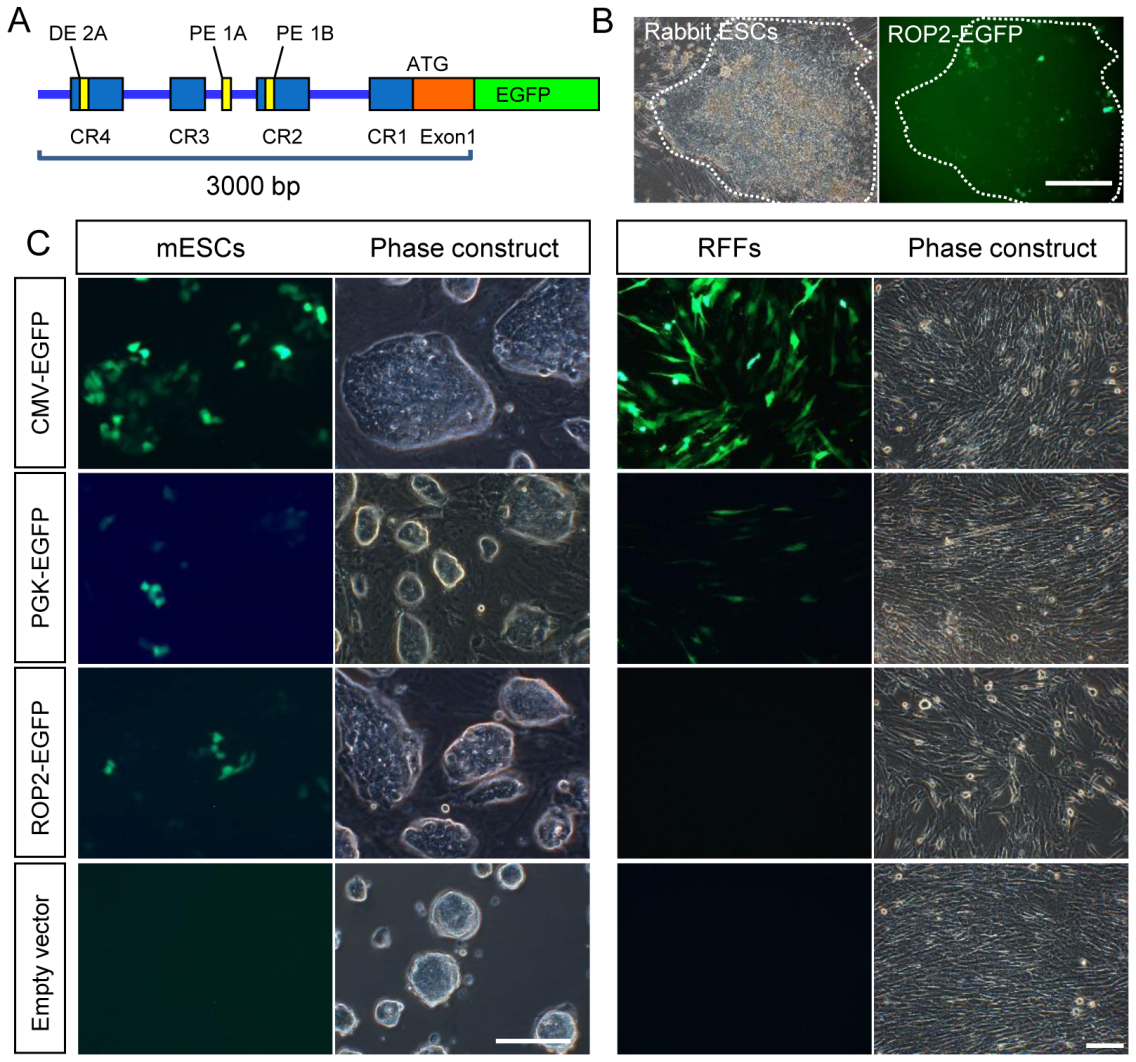
Construction of a rabbit Oct4 promoter-based EGFP vector. (A) Diagram of the pROP2-EGFP vector containing a 3.0 kb rabbit Oct4 promoter followed by EGFP in our experiments. (B) Partial EGFP expression in Rb-ESCs after transfection with pROP2-EGFP. (C) EGFP images after the transfection of CMV-GFP, PGK-EGFP, pROP2-EGFP and empty vector into murine ES cell line (R1) and rabbit fibroblasts (RFFs). Scale bars = 50 µm.

The sequences of the rabbit Oct4 gene and its promoter have been previously reported [20]. The rabbit Oct4 promoter contains four conserved regions (CR1, CR2, CR3 and CR4) and two enhancers. We selected a 3.0 kb-long sequence that contains the four conserved regions, the distal enhancer (DE), the proximal enhancer (PE) and the first exon as the promoter (Fig. 1A). The promoter was amplified from a New Zealand white rabbit by genomic PCR and then cloned into the upstream of the EGFP-coding region to generate the pROP2-EGFP vector.

We used the Rb-ESCs to test the specificity of the pROP2-EGFP vector by cell transfection and found that EGFP fluorescence was partly expressed in the colonies (Fig. 1B). We separately transferred the CMV-EGFP, PGK-EGFP and pROP2-EGFP vectors into R1 ESCs and ordinary rabbit fetal fibroblasts (RFFs) to further verify the effectiveness of the pROP2-EGFP vector. EGFP was highly expressed under the control of the CMV promoter (Fig. 1C, first row) and moderately expressed under the control of the PGK promoter (Fig. 1C, second row). By contrast, EGFP from pROP2-EGFP was activated with moderate levels in R1 ESCs but not in RFFs (Fig. 1C, the third row).

### Establishment of Oct4-EGFP transgenic RFFs

**Figure 2:**
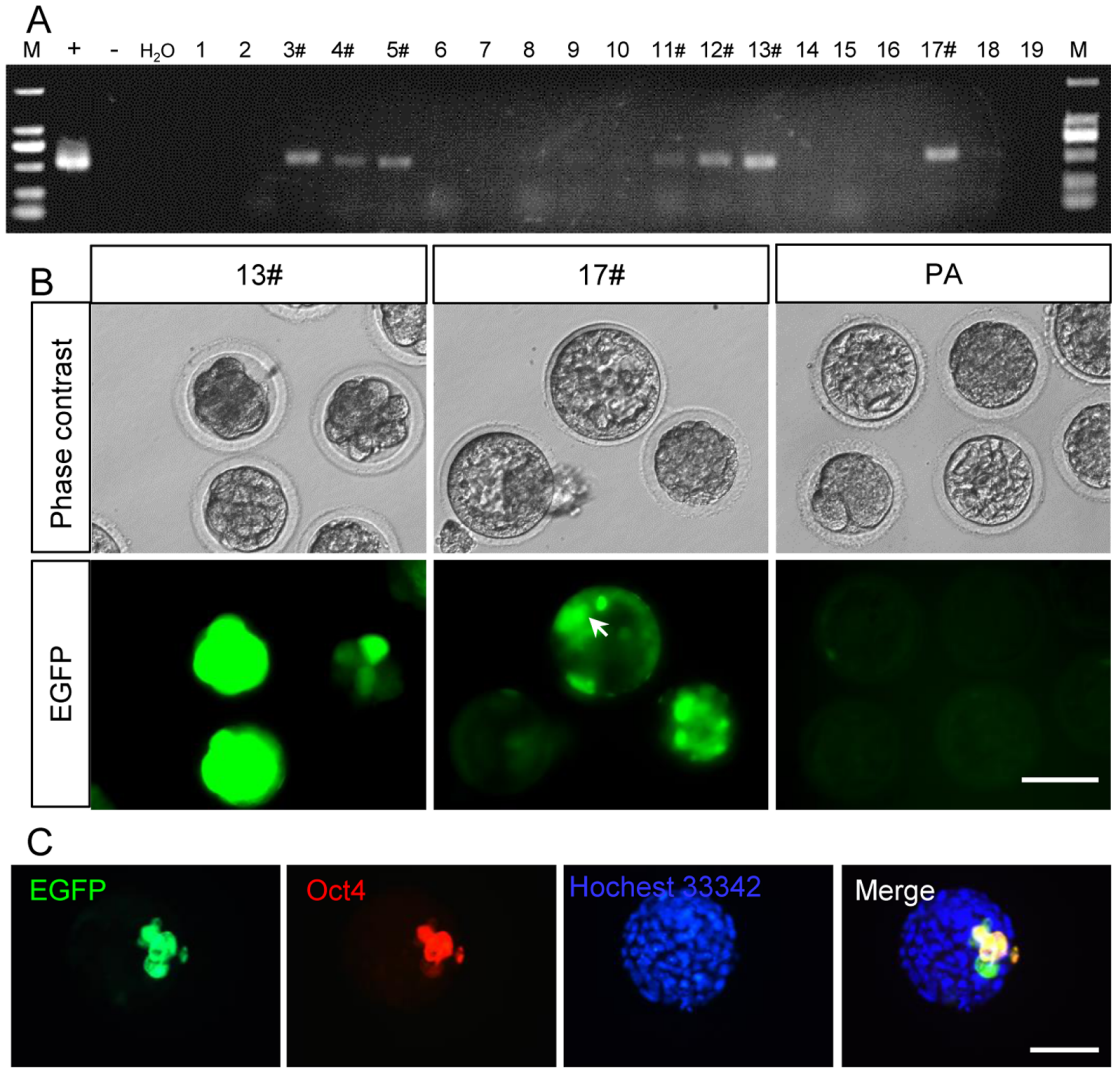
Selection of RFF donor cells stably transfected with pROP2-EGFP for nuclear transfer. (A) PCR analysis of rabbit fibroblasts stably transfected with pROP2-EGFP. (+: positive control; –: negative control; 1–19: G418-resistant clones; 3#, 4#, 5#, 11#, 12#, 13# and 17#: positive transgenic clones). (B) EGFP expressed in NT embryos using 13#, 17# fibroblasts as nuclear donors, while parthenogenetic blastocysts did not express EGFP. The arrow points ICM. PA: parthenogenetic embryos. (C) Immunoflorescent assay showed that EGFP co-expressed with Oct4 in the ICM from NT embryos at the late blastocyst stage. Scale bars = 100 µm.

RFFs were transfected with the pROP2-EGFP vector by electroporation and selected using G418 for 2 weeks. A total of 135 cell clones were harvested for passage, and 19 cell lines were established. Seven cell lines (marked 3#, 4#, 5#, 11#, 12#, 13# and 17#) were positive for pROP2-EGFP vector integration as confirmed by genomic PCR (Fig. 2A). We selected 17# fibroblasts as nuclear donors for SCNT. The cleavage rate of oocytes was 90.9% (40/44) at 24 hours after activation, and the blastocyst rate reached 40.9% (18/44) (Table 1), indicating that the in vitro development of nuclear-transferred embryos was not affected by the integration of the pROP2-EGFP vector. The morulae and blastocysts were examined via fluorescence microscopy. EGFP expression was found in the morulae and blastocysts (8/18, 44.4%) but not in the parthenogenetic (PA) counterpart embryos. Other transgenic fibroblasts, such as 13# fibroblasts, were also used for SCNT. EGFP was observed when the embryos developed to morula and ICM of blastocysts. Trophoblast cells also expressed weak EGFP fluorescence at the early blastocyst stage (Fig. 2B). Immunostaining results showed that EGFP and Oct4 were co-expressed in the ICM of the late blastocyst stage, while both were inactivated in trophoblast cells (Fig. 2C). These results indicated that the Oct4 promoter was activated when somatic cells with Oct4 promoter EGFP vector were reprogrammed into pluripotent cells by the cytoplasm of oocytes through nuclear transfer.

**Table 1:** In vitro development of SCNT embryos.

Donor cell lines	reconstructed embryos	fused couplets	cleaved embryos (%)	blastocysts (%)	EGFP-positive blastocysts (%)
17#-RFF	68	44	40 (90.9%)	18 (40.9%)	8 (44.4%)
wt-RFF	30	26	24 (92.3%)	11 (42.3%)	0 (0%)
PA(no donor cells)	11	–	11 (100.0%)	8 (72.7%)	0 (0%)

17#-RFF: 17# pROP2-EGFP transgenic rabbit fibroblasts; wt-RFF: wide type rabbit fibroblasts; PA: parthenogenetic embryos.

### Analysis of nuclear transfer fetuses

**Figure 3:**
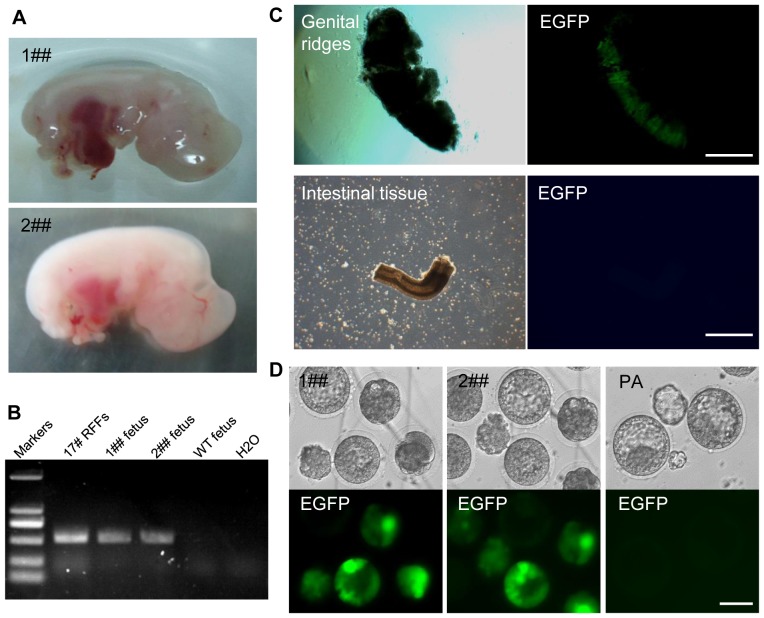
Analysis of NT fetuses. (A) Two cloned fetuses (named 1## and 2##) derived from 17# transgenic clones by SCNT at 15 days after transplantation. (B) Genomic PCR analysis of fetuses 1## and 2## stably transfected with pROP2-EGFP. NC: negative control. (C) EGFP expressed in genital ridges isolated from 2## fetus but not in other tissues, such as intestinal tissues. Scale bars = 1 mm. (D) EGFP reactivated in morulae and blastocysts after the second SCNT was performed using Oct4-EGFP transgenic fibroblasts isolated from fetus 1## and 2## as donors. Parthenogenetic blastocysts did not express EGFP. Scale bars = 100 µm.

Forty-eight 17# nuclear-transferred embryos were transferred into three surrogate rabbits at 1 day after activation. At 15 days after embryo transfer, two fetuses (marked 1## and 2##) were retrieved from two surrogates by caesarean section (Fig. 3A). Genomic PCR analysis confirmed that both of these fetuses were integrated with the exogenous pROP2-EGFP vector (Fig. 3B). Genital ridges were collected and observed under a microscope. EGFP expression was found in the genital ridges of both fetuses, but not in other tissues, such as intestinal tissues (Fig. 3C). Neural stem cells (Rb-NSCs) and RFFs were isolated from the fetuses of the two transgenic rabbits. Fibroblasts were then used as donor cells to perform a second round of nuclear transfer. EGFP expression was also found in the morula and ICM of blastocysts (Fig. 3D). This result was also observed in the response of the embryos derived from the original transgenic fibroblasts. Therefore, the Oct4 promoter was reactivated.

### Generation of Rb-iPSCs from transgenic Rb-NSCs

**Figure 4:**
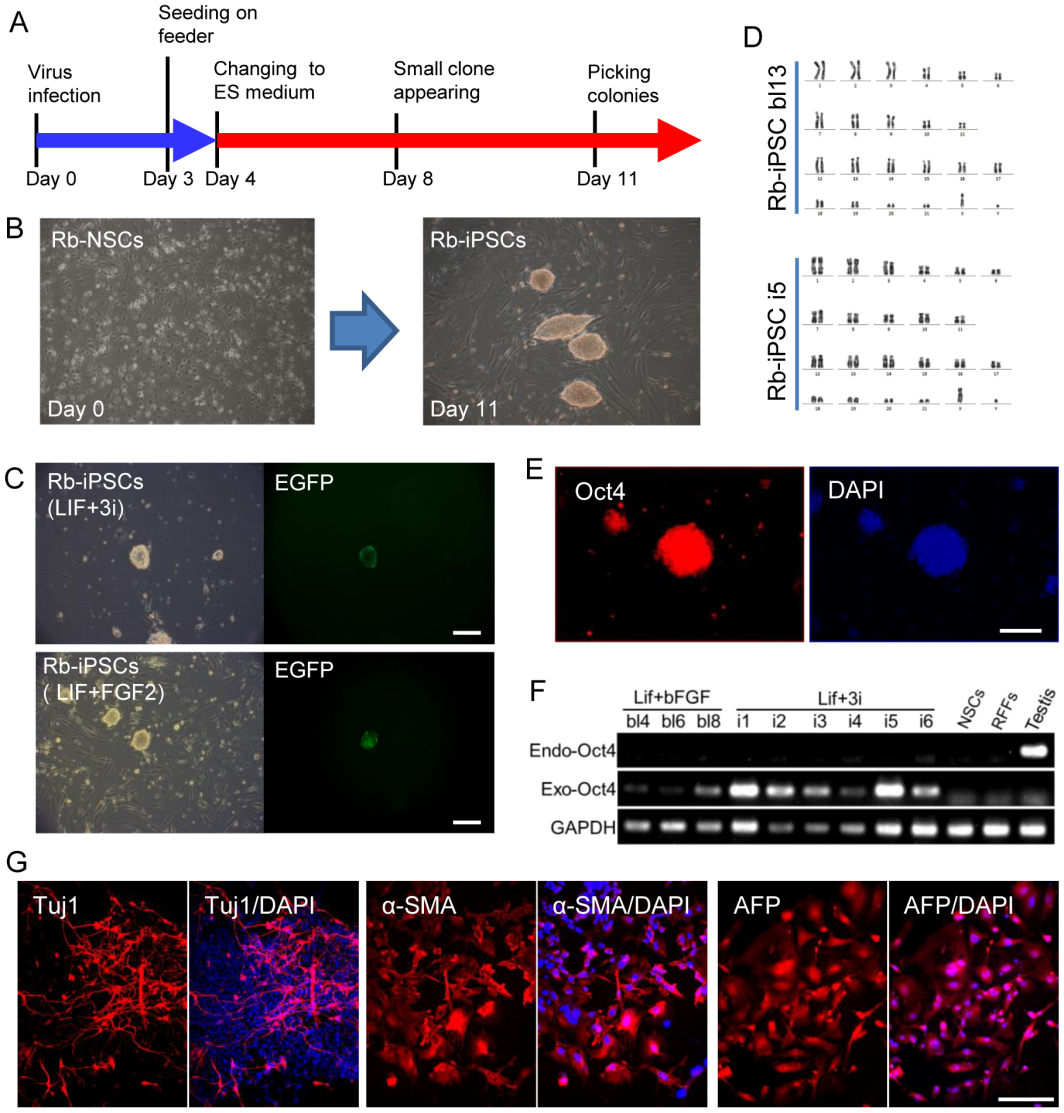
Generation of Rb-iPSCs from transgenic Rb-NSCs. (A) Experimental outline for generating Rb-iPSCs from Rb-NSCs. (B) The morphology of NSCs changed to multi-layer compacted clones after 11 days of induction using human transcription factors Oct4, Klf4, Sox2 and c-Myc. (C) EGFP activated in several Rb-iPS colonies in 3i medium (hLIF plus 3i) and BL medium (hLIF plus bFGF). (D) Rb-iPSCs (Rb-iPSC bl13 cultured in BL medium and Rb-iPSC i5 cultured in 3i medium) maintained a normal karyotype at 15 passages. (E) Immunostaining assay showed the expressed Oct4 in Rb-iPSCs. (F) RT-PCR result demonstrating that endogenous Oct4 was not expressed in all the proliferated colonies and that exogenous Oct4 was continuously expressed in Rb-iPSC colonies. (G) Expressed markers of the three germ layers after Rb-iPSCs differentiated in vitro. Tuj1 (ectoderm); AFP (endoderm); and smooth muscle actin (mesoderm). Scale bars = 50 µm.

We used Rb-NSCs isolated from the Oct4-EGFP transgenic fetuses to tentatively generate Rb-iPSCs through the infection of lentiviral vectors that encode four human transcription factors (Oct4, Sox2, Klf4 and c-Myc) (Fig. 4A). The cellular morphology changed at 4 days after infection, and iPS-like colonies were first observed after 8 days. The morphological characteristics of iPS-like colonies with multi-layer compacted cells were similar to those of mouse and rat ESCs (Fig. 4B). At 11 days to 12 days after the initial induction, EGFP expression could be detected in several iPS-like colonies (approximately 0.5% of all colonies) (Fig. 4C). The iPS-like colonies that express EGFP were harvested for further culture. EGFP expression rapidly weakened and completely disappeared at the first or second passage. These colonies can proliferate and maintain iPSC morphology in both BL and 3i media. Twenty three colonies in BL (hLIF plus bFGF) medium and 31 colonies in 3i (hLIF plus 3i) medium were passaged, and 13 colonies in BL medium and 10 colonies in 3i medium can be cultured for as long as 15 passages with normal karyotypes (Fig. 4D). Immunofluorescence demonstrated that most of the colonies without EGFP expressed Oct4 (Fig. 4E). RT-PCR analysis showed that the detected Oct4 expression was derived from exogenous Oct4 (human Oct4) instead of endogenous Oct4. These results indicated that the pluripotency of Rb-iPSCs was maintained by exogenous Oct4 and not by endogenous Oct4 (Fig. 4F).

We also investigated the in vitro differentiation capacity of the obtained Rb-iPSCs. The cells cultured undeved embryoid bodies (EBs) at 5 days to 7 days. The EBs were allowed to attach to the bottom of the culture dishes and then further cultured for 9 days to 10 days. Differentiated cells were stained with three germ layer markers, namely, Tuj1 (ectoderm), AFP (endoderm) and smooth muscle actin (mesoderm) (Fig. 4G). The differentiated cells tested positive for the three markers, suggesting that the Rb-iPSCs can differentiate into the three germ layers.

## Discussion

Oct4 is considered as an essential marker used to monitor pluripotent stem cells (e.g., ESCs, iPSC and PGCs). However, the establishment of rabbit ES and iPSCs lags far behind that of human and mouse. This drawback is principally attributed to the lack of knowledge regarding the mechanisms underlying the differentiation and reprogramming of rabbit embryos and cells. An animal model with Oct4-EGFP reporting system used to monitor the differentiating and reprogramming states similar to those in murine iPSCs and ESCs could benefit studies that focus on the differentiation and reprogramming of rabbit embryos and cells.

Although some conserved regions exist, sequences are diverse in some regions of the Oct4 promoter from different species. An Oct4 promoter from the same species appears to be more suitable for a reporter system than a promoter from different species [12], [13], [21]. The human Oct4-EGFP reporter failed to monitor pig pluripotent cells, although the murine Oct4-EGFP reporter exhibits satisfactory performance in porcine cells [11]. The expression pattern of EGFP in porcine and bovine embryos is different from that of endogenous Oct4 when a murine Oct4-EGFP construct is injected into pig and cattle zygotes, respectively [22]. The murine Oct4 promoter without PE cannot be activated in rabbit embryos [23]. To accurately monitor a specific animal species, the Oct4 promoter from the same species should be used to establish an effective Oct4-EGFP reporting system. In the present study, the rabbit Oct4 promoter was selected to monitor endogenous Oct4 expression and an applicable Oct4-EGFP reporting system was establish in rabbits.

EGFP was activated in rabbit and mouse ESCs but not in RFFs when the pROP2-EGFP vector was transfected into the rabbit ESCs, mouse ESCs and RFFs. These results confirmed the functionality and specificity of the pROP2-EGFP vector for directing EGFP expression in pluripotent stem cells but not in differentiated cells. SCNT was used to obtain pROP2-EGFP transgenic embryos and fetuses. SCNT provides an effective approach for reprogramming a differentiated cell into a pluripotent one, which can be used to clone animals when reconstructed embryos are transferred into a surrogate [24]. In the present study, SCNT was used as a rapid and simple system to verify the functionality of our rabbit Oct4-EGFP reporting system. The somatic cells integrated with the Oct4-EGFP vector were reprogrammed into NT embryos that contain pluripotent stem cells, such as morulae and ICMs with green fluorescence. This result indicated that our system can be used to monitor rabbit pluripotent stem cells.

We found that 44.4% of the reconstructed blastocysts expressed EGFP which is consistent with other reports on rabbits and pigs using Oct4-EGFP reporter systems [25], [26]. The rate of embryos expressing EGFP, instead of blastocyst rate, may represent the actual reprogramming efficiency of SCNT. Bortvin et al. has reported that SCNT embryos can efficiently develop to blastocysts [27]. However, they found that only 62% of blastocysts can express Oct4 and Oct4-related genes and that the incorrect expression of Oct4 related genes may responsible for implantation failure.

For pluripotent stem cells, two statues of pluripotency exist: the naïve state represented by mouse ESCs and primed state represented by epiblast stem cells (EpiSCs) [28]. The DE of the mouse Oct4 promoter is active in ESCs, ICM cells of blastocyst, and primordial germ cells. Conversely, the PE is effective in the EpiSCs and epiblasts of post-implantation embryos [9]. The sequence of the rabbit Oct4 promoter used in this experiment consisted of both DE and PE. Thus, the Oct4-EGFP report system established in this study can be used to verify the pluripotency of stem cells but not to distinguish naïve cells from primed cells. To establish a system reporting the whether the cells are in naive state or primed state, the Oct4 promoter without the DE or the PE should be used respectively as previously reported in mice [9].

Yin et al. [25] have recently conducted a similar study. The following are the key differences between our work and that of Yin et al. Firstly, the sequence of the Oct4 promoter in our work included not only the DE, the PE and the basic promoter region but also the whole region of the first exon to simulate endogenous Oct4 expression for the regulator may also in the exons [29]. Secondly, both studies detected EGFP expression in ICM and trophoblasts at the early blastocyst stage. However, we further found that EGFP gradually disappeared from trophoblasts and only expressed in ICM at the late blastocyst stage. Thirdly, different from Yin et al., we found that EGFP was expressed in genital ridges. This result proved that pluripotent embryonic germ (EG) cells in rabbits, similar to those in mice [21] and pigs [30], are transmitted into genital ridges.

One of the most important applications of Oct4 promoter-EGFP system is to monitor the process of reprogramming somatic cells into pluripotent cells. The somatic cells from the fetuses or rabbits should be used to conduct the reprogramming test. In contrast to Yin et al., we carried out two more tests to verify the effectiveness of the Oct4-EGFP system. In the first test, we conducted the second round of SNCT using pROP2-EGFP transgenic RFFs as nuclear donors. In the second test, Rb-NSCs derived from the transgenic fetuses were further used to generate Rb-iPSCs. The expression of EGFP was found in the resulted nuclear transfer embryos. Clearly, the reprogramming capacity of oocytes is reliable.

Two laboratories obtained Rb-iPSC colonies using a similar culture medium with growth factors LIF and bFGF [2], [31]. In the present study, we also used 3i medium aside from BL medium to establish rabbit ES cell lines to help the cells maintain their naïve status [17]. The iPS-like colonies with multi-layer compacted cells, which were similar to those of mouse and rat ESCs, were achieved using both BL and 3i media. However, only a few of the Rb-iPSCs induced with these Yamanaka's factors displayed EGFP expression. In addition, EGFP expression was transient and quickly disappeared at early passages, suggesting that the endogenous Oct4 can be reactivated in the early passages of iPS-like colonies. However, the culture system with either BL or 3i medium did not sustain endogenous Oct4 expression for a long-term culture. RT-PCR assay demonstrated that exogenous Oct4 rather than endogenous Oct4 maintained the pluripotent state of these iPSCs. Thus, we established pre-iPSCs instead of naïve or primed iPSCs, although they were able to differentiated into the three germ layers in vitro as previously reported [2],[31].

In conclusion, a rabbit Oct4-EGFP reporting system was successfully established. This system can be applied to monitor the pluripotent state of rabbit cells. Our proposed system can benefit studies on early embryonic development and on differentiating and reprogramming mechanisms of rabbit pluripotent stem cells.
